# Atrial cardiomyopathy

**DOI:** 10.1002/ehf2.15158

**Published:** 2024-11-10

**Authors:** Wojciech Kosmala, Monika Przewłocka‐Kosmala

**Affiliations:** ^1^ Department of Heart Diseases Wroclaw Medical University Wroclaw Poland

The atria carry out three distinct functions during the cardiac cycle, serving as reservoir during systole, passive conduit during early diastole and booster pump during late diastole to regulate ventricular filling. Their contribution to maintaining cardiac functional homeostasis is critical. In particular, the left atrium (LA) plays a crucial role in connecting the right ventricle and pulmonary circulation to the left ventricle, acting as a marker of left ventricular (LV) performance and actively aiding in normal heart function. Atrial cardiomyopathy (ACM) is defined as structural, functional or electrophysiological changes in the atria having the ability to induce clinically important manifestations. In addition to the involutional alterations that occur with age, various aetiologies affecting the heart can lead to ACM, including heart failure (HF), myocarditis, valvular heart disease, hypertension, diabetes, obesity, obstructive sleep apnoea, systemic inflammatory and autoimmune diseases, hormonal disorders promoting adipogenesis and others.

The aim of this editorial for the Virtual Issue of ESC Heart Failure is to present a contribution of studies published in the Journal to this important topic.

The morpho‐functional substrate accounting for ACM includes atrial enlargement, fibrosis with impaired diastolic and contractile properties of atrial myocardium, abnormal electrophysiological features favouring the development of atrial fibrillation (AF), which further aggravates atrial remodelling, and atrial endothelial dysfunction.^1^ In patients with HF, volume and pressure overload stimulate atrial wall stress and cardiomyocyte stretching, deteriorating atrial damage and propelling a vicious circle of the disease. At the ultrastructural level, ACM can be characterized by cardiomyocyte abnormalities with absence of sarcomeres, accumulation of glycogen, chromatin derangements, mitochondrial dysfunction, reactive oxygen species overproduction and flawed calcium handling, enhanced collagen deposition and fibroblast proliferation, infiltration of adipose tissue and inflammatory cells, deposition of glycosphingolipids and amyloid, alterations of excitation‐contraction coupling and autonomic nerve distribution.^1^ Thus, the atrial myopathic process is triggered and maintained by a mixture of physiological and pathological phenomena such as aging, inflammation, oxidative stress, fibrosis and atrial wall stretching. These factors are paralleled by increased neurohormonal drive involving activation of renin‐angiotensin‐aldosterone system, mitogen‐activated protein kinase pathway, transforming growth factor beta and matrix metalloproteinases, autonomic system remodelling, and secretion of adipokines regulating the fibroblastic and inflammatory milieu by epicardial adipose tissue, all of which contribute to the development of ACM.

The clinical consequence of ACM is a worsening prothrombotic state and subsequent thromboembolic complications, including stroke. This is due to atrial dilatation, impaired atrial mechanical function, concomitant AF, atrial endothelial dysfunction and the release of clot‐promoting molecules. Existing evidence indicates that the thromboembolic risk conferred by ACM extends beyond the prothrombotic effect of AF itself. Accordingly, the concept of ACM may facilitate understanding of the increased risk of stroke in patients with sinus rhythm.

From the clinical point of view, ACM cannot be considered exclusively in the context of AF and increased likelihood of thromboembolic complications. ACM represents an important component of HF pathophysiology, developing either secondary to LV dysfunction or without prior LV impairment. In the latter scenario, atrial structural and functional derangements may generate HF syndrome through impaired LA‐LV coupling, thus being the starting point of HF spiral. This refers to the proposed term of ‘LA failure’ denoting any atrial dysfunction causing impaired cardiac performance and symptoms and worsening quality of life or life expectancy, in the absence of significant valvular or ventricular abnormalities.^2^ Existing data indicate that in some cases, HFpEF may be the consequence of LA failure, which can be an early driver and key contributor to the development of HF symptoms.

Accumulating evidence suggests that LA functional parameters may be superior to LA size in characterizing ACM. Notably, LA strain is an important biomarker even in the absence of LA dilatation, with changes in LA deformation preceding alterations in atrial volumetric indices. LA strain reflects both LA and LV function, identifies LV filling pressure elevation and serves as a relevant prognosticator in HF.^3^

The crucial role of LA deformation in the pathophysiology of HF was highlighted in the paper by Maffeis et al.^4^ The authors demonstrated that LA reservoir strain was the strongest predictor of reduced exercise capacity across all HF categories, incremental over clinical and demographic factors, and other echocardiographic variables, including LV global longitudinal strain and LV ejection fraction. The cut‐off value of LA reservoir strain <23% was characterized by very high sensitivity (96%) in identifying a severely impaired peak VO_2_ < 14 mL/kg/min.

The paper by Bo et al. revealed a significant association between LA strain and major adverse cardiac events, including cardiovascular death and HF hospitalization in HFrEF with both ischemic and non‐ischemic aetiology. LA strain provided incremental prognostic information over traditional predictors (age, BMI, co‐morbidities, renal function and natriuretic peptides), and patients in the lower LA strain tertile (cutpoint <8%) had a significantly increased risk of adverse outcome irrespective of the presence of late gadolinium enhancement—a marker of fibrosis.^5^

Recent evidence linking isolated functional tricuspid regurgitation and HFpEF through the pathophysiology of ACM has gained support from the work of Seo et al.^6^, ^7^ The decline in LA reservoir strain after surgical correction of functional tricuspid regurgitation was the only echocardiographic determinant of all‐cause mortality and a predictor of refractory AF.

LA strain analysis can be useful in diagnosing the presence of ATTR‐cardiac amyloidosis in patients with aortic stenosis. ROC analysis demonstrated that peak LA strain rate was a better predictor of amyloidosis than the apical sparing pattern of LV strain (AUC 0.79 vs. 0.69, respectively).^8^ Pathophysiologically, this finding is consistent with the promotion of ACM by amyloid deposits.

The use of conventional parameters of LA function revealed that the prognostic value of LA functional remodelling in HFpEF may differ depending on the presence of AF. LA ejection fraction <40% was associated with a composite endpoint of HF hospitalization and cardiovascular death only in patients with sinus rhythm but not with AF, providing a sensitivity of 90% and specificity of 65% in predicting adverse outcome.^9^

Atrial functional mitral and tricuspid regurgitations are atrial remodelling‐associated valvular abnormalities that may worsen atrial disturbances and promote AF. Masuda et al. demonstrated that catheter ablation of AF reduced atrial functional mitral regurgitation, and this effect was likely to be mediated by LA reverse remodelling, as assessed by a post‐procedural decrease in LA volume index (−11.4 mL/m^2^ vs. −2.3 mL/m^2^ in the subsets with and without mitral regurgitation improvement, respectively). Regression of mitral regurgitation was associated with a less frequent composite of all‐cause death and HF hospitalization. Patients with extensive LA low‐voltage areas >20 cm^2^, believed to have more LA fibrosis, exhibited less LA reverse remodelling and a smaller reduction in the severity of mitral regurgitation.^10^ The positive effect of catheter ablation of AF on LA size regression was also reported in the paper by Lee.^11^

Given the relevant role of ACM in the pathogenesis of HF and AF, LA remodelling has become a target for pharmacotherapies. Sun et al. demonstrated that the use of ARNI was superior to ACEI and ARB in attenuating LA dilatation in HFrEF and ablation‐treated AF.^12^, ^13^ Patients with mildly increased LA size (LA volume index 29–34 mL/m^2^) receiving ARNI showed a survival benefit over those on ACEI/ARB, which supports the notion of early commencement of sacubitril/valsartan therapy when LA remodelling is less advanced.^12^

A beneficial effect of dual SGLT1 and SGLT2 inhibition on LA function was noted in HFpEF.^1^, ^14^

Septal myectomy, relieving LV outflow tract obstruction in hypertrophic cardiomyopathy improved both structural (LA volume) and functional (LA strain) components of ACM.^15^ The effects of mavacamten—a cardiac‐specific myosin inhibitor are expected to be assessed also in relation to LA remodelling.
